# The COVID Psychosocial Impacts Scale: A Reliable and Valid Tool to Examine the Psychosocial Impacts of the COVID-19 Pandemic

**DOI:** 10.3390/ijerph20115990

**Published:** 2023-05-29

**Authors:** Sandila Tanveer, Philip J. Schluter, Ben Beaglehole, Richard J. Porter, Joseph Boden, Ruqayya Sulaiman-Hill, Damian Scarf, Shaystah Dean, Fatima Assad, Mahammad Abul Hasnat, Caroline Bell

**Affiliations:** 1 Department of Psychological Medicine, Christchurch Campus, University of Otago, Christchurch 8011, New Zealand; 2 Te Kaupeka Oranga|Faculty of Health, Te Whare Wānanga o Waitaha|University of Canterbury, Christchurch 8041, New Zealand; 3 School of Clinical Medicine, Primary Care Clinical Unit, The University of Queensland, Brisbane, QLD 4072, Australia; 4 Department of Psychology, University of Otago, Dunedin 9016, New Zealand; 5 Department of Psychological Medicine, Wellington Campus, University of Otago, Wellington 6021, New Zealand; 6 Department of Psychiatry, HITEC Institute of Medical Sciences, Taxila 47078, Pakistan; 7 Department of Education, Milestone College, Dhaka 1230, Bangladesh

**Keywords:** COVID-19, psychosocial impacts, psychological distress, wellbeing, reliability, validity

## Abstract

This paper reports on the development and validation of the COVID Psychosocial Impacts Scale (CPIS), a self-report measure that comprehensively examines both positive and negative psychosocial impacts from the COVID-19 pandemic. This is the first part of the program of work in which the CPIS was administered and compared with a measure of psychological distress (Kessler Psychological Distress Scale, K-10) and wellbeing (World Health Organization Well-Being Index, WHO-5). The data were obtained online in 2020 and 2022 at two distinct time points to capture different exposures to the pandemic in the New Zealand population to a non-representative sample of 663 and 687 adults, respectively. Two hundred seventy-one participants took part in both surveys. Findings indicate a unidimensional structure within CPIS subscales and inter-relatedness among CPIS stress-related subscales. The scatter plots and correlation matrix indicate CPIS having a positive moderate correlation with K10 and a negative moderate correlation with WHO-5, indicative of construct validity. The paper outlines contextual factors surrounding CPIS development and makes suggestions for future iterations of CPIS. Further work will examine its psychometric properties across cultures.

## 1. Introduction

The novel coronavirus disease 2019 (COVID-19) pandemic has exposed people to significant and prolonged stress [1,2]. The myriad of primary stressors includes personal and familial health consequences of infection, together with social and financial impacts of the ongoing public health restrictions, e.g., lockdowns, social distancing requirements, travel bans, and vaccination mandates. These psychosocial impacts were amplified by uncertainty relating to new variants of COVID-19, the limited effectiveness of public health restrictions, and political responses. What was happening amid increased societal divisions and polarisation around vaccination and mask mandates, and associated additional stresses (e.g., people losing jobs, verbal abuse of vaccinators, and staff checking vaccination passports) [3]. Those restrictions have largely been removed, although uncertainty about future variants remains. 

No one is exempt from exposure to pandemic-related stressors but some segments of society (e.g., health workers, law enforcement, providers of essential goods/services, and at-risk populations) are more vulnerable to these stressors than others [4]. Some of the psychosocial impacts of COVID-19 were anticipated from the start of the pandemic [5]; however, the fluid nature of the pandemic globally, both within and across contexts and the varied responses, has made the assessment and cross-cultural comparison of the psychosocial impacts of COVID-19 extremely challenging. 

Different approaches have been utilized to examine the psychological and social impacts of the COVID-19 pandemic. These include employing cross-sectional [6,7,8,9,10,11,12] and longitudinal [13] study designs with a representative [6,7,8] and convenience samples [9] of the general population, those in specific work roles (especially healthcare) [10,11], or those having a previous history of mental disorders [12,13]. Most studies have employed standardized self-report measures of wellbeing, psychological distress, depression, anxiety, and post-traumatic stress disorder (PTSD). These studies have generally reported negative psychosocial impacts, for example, psychological distress [8,14], alcohol abuse [14], family harm [8], and suicidality [8,14,15]. Some studies have used standardised instruments across countries to obtain comparable data sets [6,7,10]. Although the use of pre-existing standardised measures also allows for pre-pandemic comparisons, these measures do not specifically examine the impacts of COVID-related stressors. 

As a result, there have been recommendations for novel self-report measures to be used alongside established psychometric measures [16,17]. Several scales have been developed to this end, which vary in focus and length. These include those assessing the impacts of the COVID-19 pandemic and public health restrictions on physical health, employment and finances, family and social disruption; and measures of psychological distress, loneliness, anxiety, depression, and PTSD (see [17] for a summary of these measures). While few scales attempt to capture the psychosocial impacts of the COVID-19 pandemic (for example, CRISIS assesses COVID stress on key domains namely COVID exposure, COVID worries, life changes, mood states, daily behaviors) [18], they frequently reflect the context and country in which they were developed, limiting their more general applicability. Other scales have a more specific focus rather than assessing the wider impacts of the pandemic (for example, the Fear of COVID-19 scale [19], the Coronavirus Anxiety Scale [20], and the COVID-19 Anxiety Scale [21]). With the exception of the Fear of COVID-19 scale, most of these novel scales lack detailed validation studies. 

Importantly, the potential positive impacts of the pandemic were also recognised [22] but very few empirical studies have been undertaken to examine the positive psychosocial impacts of the pandemic. Jenkins et al. [23] and Beaglehole et al. [24] reported data from a demographically representative sample of New Zealanders. Jenkins et al. described the experience of the silver linings (positive impacts) resulting from the COVID-19 pandemic and reported positive impacts in two-thirds of their sample. They also reported two overarching themes of positive psychosocial impacts, including surviving (coping well, meeting basic needs, and maintaining health) and thriving (self-development, reflection, and growth) [23]. Beaglehole et al. examined lockdown experiences, wellbeing, and key sociodemographic determinants; and reported excellent wellbeing in 9% of their sample during the lockdown. They emphasised the need to assess a positive wellbeing to gain a comprehensive understanding of the psychosocial impacts of the COVID-19 pandemic [24]. 

The COVID Psychosocial Impacts Scale (CPIS) was therefore developed to address some of the issues identified above. The CPIS was developed by academics and practitioners in the field of psychology, psychiatry, epidemiology, and public health in the very early stages of the pandemic in New Zealand when we had a strict lockdown and very few cases. This situation did not change substantively until early 2022 when the Omicron variants arrived with higher infection rates, despite a high vaccination rate. We aimed to develop a measure for use in adults (18 years and above) that comprehensively assessed the impacts of the COVID-19 pandemic, including the adverse personal, social, and economic consequences that followed and recognised the positive outcomes which may have occurred. Ideally, the items must be derived based on prior research. However, it was the first historical occurrence of a widespread novel virus during a period in history where we have in place the tools to try to understand the stress caused by it. Notably, at the time of designing the CPIS, there were no established measures for assessing COVID-related stresses. As a consequence, we used an empirically driven approach to item and scale construction informed by our experience of developing a nuanced model for measuring exposure to the Canterbury earthquakes [25,26], in which the domains were modelled on a modified version of the Social Readjustment Scale [27]. In this approach, participants are asked to indicate the life event they have experienced and subsequently report the resultant level of stress owing to that life event. This approach allows us to work out the relative “weights” of different types of stresses and also shows combined stress load. Importantly, the relevant literature [4,5,6,8,10,11,12,13,16,17] and existing pandemic scales [18,19,20,21] were consulted in the process of scale construction. The CPIS applicability for use in a range of countries and cultures was also considered in the scale development. 

There were three steps to scale construction, namely item development (identification of domain, item generation, content validity); scale development (pre-testing of items, survey administration, item reduction, extraction of factors); and scale evaluation (reliability, construct validity) [28]. The items were empirically derived from clinical experience in which participants were asked to indicate the resultant level of stress to a pandemic-related life event. Notably, the existing pandemic-related scale that examined the psychosocial impacts [18] was consulted in the process of item generation and identification of domains. Questions examining the positive consequences of the COVID-19 pandemic were also included. The CPIS was refined using the ‘group mind’ process [29], which asks colleagues including researchers based on personal contacts from a range of countries and cultures (Bangladesh, Ghana, and Pakistan) to review and rigorously critique a draft of the questionnaire, with iterative improvements made based on their comments. These specific low- and middle-income countries faced the substantial burden of COVID-19 (unlike New Zealand) and also vary in socio-economic context to New Zealand where the CPIS was developed. Collectively, this provided sufficient context to design a widely applicable measure. This was done to ensure content validity and applicability of newly developed CPIS in varying contexts. 

In the scale development phase, we pre-tested the revised questionnaire on a small sample of the general public and further modified it to address respondents’ feedback. The CPIS was then administered to a non-representative New Zealand population at two distinct time points (2020, 2022) using an online survey to examine the psychometric properties of the newly developed scale. This paper reports on the item reduction, reliability analysis, factor extraction, and construct validity of the newly developed CPIS and its comparison with standardized self-report measures of psychological distress (Kessler Psychological Distress Scale, K10) [30] and wellbeing (World Health Organization Well-Being Index, WHO-5) [31]. Importantly, the psychometric study was carried out in New Zealand at two distinct time points to capture different exposures to the pandemic in the New Zealand population: (1) in 2020 (survey 1) when the pandemic was well-controlled and there was no reported community transmission of COVID-19; and (2) in 2022 (survey 2) when approximately 35% of the population had had a reported confirmed case of COVID-19 and nearly 2800 deaths had resulted [32]. At the time of both surveys, there was no lockdown in place across New Zealand, but the conditions on the ground changed considerably from successfully suppressing COVID-19 for two years to a reduced restriction due to a high vaccination rate, followed by an infection increase in 2022. The same set of items has been used in both surveys, except three items that were modified owing to psychometric and pragmatic considerations reflecting the process of constant improvement (for details see Table 1 and Section 3.1). This is the first part of the program of work and further work will examine its psychometric properties for cross-cultural use after the data acquisition is complete.

## 2. Materials and Methods

### 2.1. Participants

Participants were recruited using the Department of Psychology Research Participation Pool (University of Otago, New Zealand) comprising students and the general population using a convenient sampling from a non-representative New Zealand population.

In survey 1, six hundred sixty-three participants completed the survey. The median age range of respondents was 40 to 49 years. There were more female participants (75%) than males and most participants identified their ethnicity as New Zealand European (88%). Participants were living either with family (47%) or a partner/spouse (30%), and the majority (88%) experienced no change in their living arrangements since COVID-19. Most participants (76%) reported spending less than an hour a day seeking information about COVID-19. Participants mainly accessed information related to COVID-19 from the news (91%), health websites (62%), and Facebook/Twitter (40%). The majority of participants (90%) had experienced at least one prior (i.e., before the pandemic) traumatic event, most commonly a natural disaster (34%) and childhood trauma before the age of 16 years (25%). Two hundred six participants (31%) had a COVID-19 test and only one participant was diagnosed with COVID-19. Nineteen (3%) participants had at least one family member who had been diagnosed with COVID-19. Seven had 1 family member, six had 2 family members, two had 3 family members, one had 4 family members, one had 5 family members, and two had 6 family members diagnosed with COVID-19. Of these, one experienced 1 bereavement and another experienced 3 bereavements. No participants reported a family member who was COVID-19 positive at the time of data collection of survey 1. 

In survey 2, six hundred eighty-seven participants completed the survey. The median age range of respondents was 50 to 59 years. There were more female participants (53%) than males and most participants identified their ethnicity as New Zealand European (89%). Participants were living either with family (36%) or a partner/spouse (44%) and the majority (84%) experienced no change in their living arrangements since COVID-19. Most participants (88%) reported spending less than an hour a day seeking information about COVID-19. Participants mainly accessed information related to COVID-19 from the news (83%), health websites (66%), and Facebook/Twitter (20%). Most participants (56%) experienced at least one prior (i.e., before the pandemic) traumatic event, most commonly a natural disaster (36%) and childhood trauma before the age of 16 years (22%). Five hundred fifty-six (81%) had a COVID-19 test. Five hundred forty (79%) participants had at least one family member who had been diagnosed with COVID-19, with 167 participants reporting having two or more family members diagnosed. Of these, five participants experienced 1 bereavement. Thirty (4%) participants reported a family member who was COVID-19 positive at the time of data collection of survey 2. See Appendix A for a detailed demographic profile of the participants. 

### 2.2. Measures

#### 2.2.1. Sociodemographic Information

Sociodemographic information collected included: age, gender (male, female, gender diverse), marital status, country of birth, years living in New Zealand, New Zealand citizenship or visa status, residing town, ethnicity, education, work status, occupation, annual family income, and prior exposure to traumatic events. Ethnicity was collected using the NZ Census question on ethnicity, which allows individuals to specify multiple ethnic groups [33].

#### 2.2.2. COVID Psychosocial Impacts Scale (CPIS)

The CPIS includes 50 items that examine the psychosocial impacts of the COVID-19 pandemic across 13 key domains. These key domains are distributed across 5 core subscales (personal stresses (personal impacts, changes to daily routines and behaviours, and exposure to information about COVID-19); social stresses (family impacts, living arrangements, and relationships); financial stresses (work and COVID-19, employment, and income); perception of overall life stresses pre/post COVID-19; and positive impacts (silver linings)) and 2 optional subscales (education-related stresses, faith-related stresses) (See Table 1). The optional subscales could be skipped by participants if not applicable. 

Of the 50 items, 42 examine the exposure to, and amount of stress experienced in response to pandemic-related life events on a 6-point rating scale (0 = No exposure to the life event, 1 = Yes exposed to the life event but caused no stress at all, through to 5 = Yes exposed to the life event and caused a lot of stress). This approach of calculating a “composite” indicator has been adopted from an established scale of life stress from the Christchurch Health and Development Study [25,34,35]. In the perception of the overall life stress pre/post COVID-19 subscale, four items ask about the perception of stress (1 = Considerably less stressed, 5 = Considerably more stressed) and psychological wellbeing (1 = Much better, 5 = Much worse) on a 5-point rating scale. The last subscale ‘silver lining’ examined the potential positive impacts of the COVID-19 pandemic through four items that asked participants to specify the positive consequences they may have experienced since the COVID-19 pandemic on a 5-point rating scale (1 = Not at all, 5 = A great deal). The items in the silver lining subscale are different from other positively phrased items (e.g., items 29, 31, 34, 36, 39, and 41) as it more generally asks to reflect any positive consequences they may have experienced since the COVID-19 pandemic, and not stresses in response to a particular pandemic-related life event. Reverse scoring was carried out for the positive impacts subscale. The CPIS composite scores can be calculated based on a linear combination of scores by items on the core subscales, where the data on optional subscales can be treated as a sub-sample.

#### 2.2.3. Established Psychometric Measures

##### Kessler Psychological Distress Scale (K10)

The Kessler Psychological Distress Scale (K10) is a 10-item scale measuring non-specific symptoms of anxiety and depression over the previous four weeks on a 5-point Likert scale (1 = None of the time, 5 = All the time) [30]. Scores are reported in a 10 to 50 range, where respondents with a score of below 20 are likely to be well, 20 to 24 are likely to have a mild mental disorder, 25 to 29 are likely to have a moderate mental disorder, and participants with a score of 30 and over are likely to have a severe mental disorder [36].

##### World Health Organization Well-Being Index 5 (WHO-5)

The World Health Organization Well-Being Index 5 (WHO-5) is one of the most widely used scales for assessing subjective psychological wellbeing [31]. It contains five positively phrased items, with respondents rating each statement for the previous two weeks. Scores range from 0 to 25, which are then multiplied by 4, resulting in 100 indicating the highest subjective wellbeing. A cut-off score of 50 and below is generally used to screen for clinical depression [37].

### 2.3. Procedure

The survey was fielded on the Qualtrics XM Platform and was designed to be compatible with a mobile phone, tablet, or computer access. It was designed to take 15 to 25 min to complete the survey. Participants were asked to read the information sheet and provide online consent before proceeding with the survey (which included the CPIS and the other measures described above). For survey 1, data were collected from the 13 October to 9 November 2020 (4 weeks); at this time, 1872 confirmed cases and 25 deaths in New Zealand were attributed to COVID-19 [32]. For survey 2, data were collected from the 2 September to 26 September 2022 (3 weeks and 4 days); by this time, 1,746,460 confirmed cases and 2799 deaths due to COVID-19 had been recorded [32]. The participation was voluntary, and no incentive was provided.

### 2.4. Statistical Analyses

Data were analysed descriptively and analytically using SPSS V26.0 (IBM, Armonk, NY, USA). The reliability of the CPIS subscales was analysed using item-total correlations and Cronbach’s α with a minimum acceptable value of 0.30 and 0.70, respectively [38]. The factor structure was examined using exploratory factor analysis on the subscale of CPIS. Further analyses were carried out using scatter plots and correlation matrix (Pearson’s *r* correlation) on CPIS and its subscales to examine construct validity with K10 and WHO-5. Additional analyses were undertaken on subsamples who participated in both surveys and on subsamples who answered on one of the optional subscales (education-related stresses, faith-related stresses). 

## 3. Results

In survey 1, a total of 1489 prospective participants were contacted with an explanation of the study, a participant information sheet, and a link to the survey. A total of 718 (48%) participants consented out of 1489 prospective participants. The non-completion rate, defined as those who started but did not complete the survey before the cut-off time, was 8% (n = 50), leaving complete responses from 663 (92%) participants. The median time taken for participants to complete survey 1 was twenty minutes (interquartile range = 15, 31 min). In survey 2, a total of 1211 prospective participants were contacted with an explanation of the study, a participant information sheet, and a link to the survey. A total of 751 (62%) participants consented out of 1211 prospective participants. The non-completion rate was 8% (n = 64), leaving complete responses from 687 (92%) participants. The median time taken for participants to complete survey 2 was nineteen minutes (interquartile range = 14, 28 min). Two hundred seventy-one participants took part in both surveys.

### 3.1. Reliability and Factor Structure

The item-total correlations and Cronbach’s α were computed for individual subscales to examine the homogeneity of the items within the subscales. Items with item-total correlations below 0.3 were removed from the analysis. In survey 1, the items removed due to low-item-total correlations included item 2 (“*Have you had a COVID-19 test?*”); item 6 (“*Do you feel any family member is at risk of being exposed to someone with COVID-19 through their work?*”); item 7 (“*Has any family member had a COVID-19 test?*”); item 12 (“*Are you considered an essential worker (e.g., in healthcare, law enforcement, emergency services, provider of essential goods/services)?*”); item 13 (“*Do you have a close friend or family member who is an essential worker?*”); and item 16 (“*Had access to adequate computing facilities (e.g., personal laptop or computer, internet, etc.)*”). The resultant reliability score for each subscale was above 0.70 (personal stress (*r* = 0.75), social stress (*r* = 0.74), financial stress (*r* = 0.79), perception of overall life stress pre/post COVID-19 (*r* = 0.86), positive impacts (*r* = 0.80), faith-related stress (*r* = 0.89), and education-related subscale (*r* = 0.75)) in survey 1. 

In survey 2, the same set of items was administered with three exceptions (items 2, 3, and 7) which were modified owing to psychometric and pragmatic considerations (See Table 1). The items taken out from analysis due to low-item-total correlations in survey 2 included item 13 (“*Are you considered an essential worker (e.g., in healthcare, law enforcement, emergency services, provider of essential goods/services)?*”); item 14 (“*Do you have a close friend or family member who is an essential worker?*”); item 28 (“*Has your household needed to seek help from a food bank or other charitable organisation?*”); and item 50 (“*Led to an increased connection to my faith*.”). The resultant reliability score for each subscale was above 0.70 (personal stress (*r* = 0.77), social stress (*r* = 0.80), financial stress (*r* = 0.83), perception of overall life stress pre/post COVID-19 (*r* = 0.89), positive impacts (*r* = 0.71), faith-related stress (*r* = 0.86), and education-related stress (*r* = 0.82)) in survey 2.

Exploratory factor analysis carried out using the varimax (orthogonal) rotation method on CPIS five core subscales identified two factors with an eigenvalue above one in both surveys (See Table 2). Factor 1 (α = 0.75 in survey 1 and α = 0.76 in survey 2) comprised personal stress, social stress, financial stress, and perception of overall life stress pre/post COVID-19 subscales with factor loadings above 0.30. The positive impacts subscale was the only subscale that contributed to Factor 2 in survey 1, whereas the perception of overall life stress pre/post COVID-19 and positive impacts subscales both contributed to Factor 2 (α = 0.12) in survey 2, with factor loadings above 0.30. Similar exploratory factor analyses were carried out including each of the optional subscales (education-related stress, faith-related stress), which identified two factors with an eigenvalue above one (See Appendix B). Only Factor 1 had an α-value above 0.70 in both surveys and it includes personal stress, social stress, financial stress, perception of overall life stress pre/post COVID-19, and each of the optional subscales with factor loadings above 0.30. 

### 3.2. Descriptive Statistics

Descriptive statistics were calculated for participants who responded to CPIS core subscales first and then for participants who responded on one of the optional subscales (education-related stress, faith-related stress) (See Table 3). An overall increase can be seen in the CPIS scores in survey 2 compared to survey 1. The mean score from K10 was below the cut-off score of 20, suggesting that participants were likely to be well. The mean score on the WHO-5 was above the cut-off of 50, suggesting the overall wellbeing of participants. The only exception was in survey 1 where the mean score on the WHO-5 was below the cut-off of 50 for participants who answered on education-related stress and faith-related stress subscales, suggesting poor wellbeing. The descriptive statistics for participants who responded on both optional subscales of CPIS are summarized in Appendix C.

**Figure 1:**
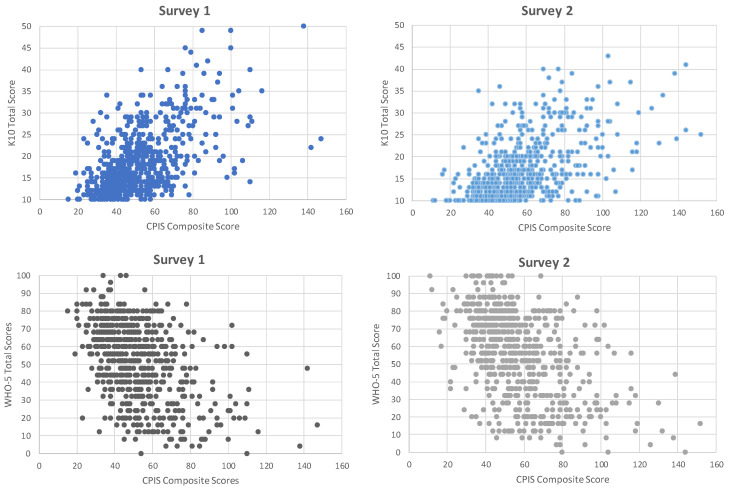
Scatter Plot Showing Correlation between COVID Psychosocial Impacts Scale (CPIS) Composite Scores with Kessler 10 (K10), and World Health Organization Well-Being Index 5 (WHO-5).

The relationships between the CPIS composite scores and K10 or WHO-5 were examined using scatter plots which showed a positive linear correlation between the CPIS composite score and K10, and a negative linear correlation between CPIS and WHO-5 (See Figure 1).

Further analysis of this linear relationship was examined using a correlation matrix, obtaining similar findings. The CPIS composite score had a positive yet moderate correlation (*r* = 0.57 in survey 1, *r* = 0.56 in survey 2) with the K10. The CPIS composite score had a negative yet moderate correlation (*r* = −0.48 in survey 1, *r* = −0.50 in survey 2) with WHO-5 (for detail see Table 4). Similar analyses were carried out for participants who responded on one of the optional subscales, education-related stress (See Appendix D) or faith-related stress (See Appendix E), which yielded similar findings.

### 3.3. Additional Analyses on Subsample

The subsample (n = 271) who took part in both surveys was analyzed psychometrically (test-retest reliability) and descriptively (mean, SD). The test-retest reliability showed moderate correlations between the two administrations of CPIS (*r* = 0.56). An overall increase in the mean scores can be observed on the CPIS core subscales (except for financial stress and positive impacts subscales), CPIS composite scores, K10, and WHO-5 in survey 2 compared with survey 1 (See Table 5). 

## 4. Discussion

This study reports the findings of the psychometric study carried out to validate the newly developed CPIS that comprehensively assessed the impacts of the COVID-19 pandemic including the adverse personal, social, and economic consequences that followed along with the positive outcomes which may have occurred. Data were obtained in 2020 and 2022 to capture different exposures to the COVID-19 pandemic from a non-representative New Zealand sample. The main findings of this psychometric study were of a unidimensional structure within the CPIS subscales and inter-relatedness among CPIS stress-related subscales. Results showed CPIS to have a moderate negative correlation with K10 and a moderate positive correlation with WHO-5, reflecting construct validity. 

Previously, Every-Palmer and colleagues examined psychological distress and wellbeing using the K10 and WHO-5 during New Zealand’s first lockdown in April 2020 in a demographically representative sample and reported moderate stress in thirty percent and low levels of wellbeing in forty percent of their participants [8]. Although the findings of Every-Palmer and colleagues are not directly comparable, due to different sampling strategies, but collectively both studies indicate a significant increase in the level of distress compared to previous population surveys [39,40], reflecting the overall impacts of the COVID-19 pandemic. Similar findings of increased psychological distress have been reported by researchers in the US [41], Europe [42,43], and Asia [44] (see also [7]). Although the use of pre-existing standardised measures allows pre-pandemic comparisons, these measures do not specifically examine the impacts of COVID-related stressors. Hence, we recommend using pandemic-related measures in conjunction with established psychometric measures to provide further depth to the interpretation of the findings.

Importantly, this study offered a unique opportunity to conduct the survey at various exposures of the COVID-19 pandemic in New Zealand and highlighted the impacts of the fluid nature of the pandemic on the findings. For survey 1, we reported mild-to-moderate levels of pandemic-related stress on CPIS amongst our sample when there was a low prevalence and no community transmission of COVID-19 in New Zealand. In survey 2, an overall increase in scores on CPIS and its subscales (personal stress, social stress, perception of overall life stress pre/post COVID-19, positive impacts) suggests that CPIS was sensitive to change in pandemic exposure. Interestingly, the participants who took part in both surveys also showed a similar trend. This may be due to a gradual recovery from the economic problems related to lockdowns and the country being closed. 

Our findings suggest that the reliability and validity scores varied across time points. We attributed this to the various pandemic exposures and situational factors. Some of the items that were not reliable at the time of survey 1 showed acceptable reliability scores at the time of survey 2. These included item 2 (“*Have you had a COVID test?*”) and item 6 (“*Do you feel any family member is at risk of being exposed to someone with COVID-19 through their work?*”). In contrast, some of the items were reliable at the time of survey 1 but did not show acceptable reliability scores at the time of survey 2. This includes item 28 (“*Has your household needed to seek help from a food bank or other charitable organisation?*”). We argue that this is an issue of a floor effect rather than a reliability effect. Floor and ceiling effects are likely to affect some items in periods of relatively low and relatively high prevalence, but importantly no sustained floor or ceiling effects were observed over the two periods. We, therefore, argue that the reliability and validity of the CPIS were reasonably stable even though there was a difference in the levels of COVID-related stresses and morbidity between the two administrations of the questionnaire. 

We also acknowledge some key contextual factors surrounding CPIS development. The experience of the pandemic in New Zealand was different compared to many other jurisdictions due to the presence of strong temporary laws that were in place to prevent the transmission of the virus. For instance, the “essential worker” category was a legal entity that permitted working outside of the home for certain designated professions during the lockdown in New Zealand, under the strict restrictions and measures in place. This was a heterogenous group (e.g., those working in healthcare, law enforcement, emergency services, or providers of essential goods/services such as those working in supermarkets or transport) in regard to income levels and risk of exposure to possible COVID-19 infection. In both surveys, a substantial percentage of participants and their families were essential workers (see Appendix A), but items 12 and 13 assessing stress owing to being an essential worker or having a family or friend as an essential worker showed poor item-total correlations by including as part of the financial stress subscale. These low-item-total correlations could be attributed to the timing of data collection as both surveys were administered when there was no lockdown in place in New Zealand. Hence, being an essential worker did not appear to contribute to stress as these items did not show much variability in the true scores, resulting in low-item-total correlations. Considering the current COVID-19 scenario, we argue that these items will be less important in future iterations of CPIS, and hence these items would likely be reconsidered as demographic items. 

In addition, in 2020 (at the time of survey 1), the item “Have you had a COVID test?” had a ‘leaf’ question “If Yes, was the result positive?” At that time there was a low prevalence of COVID-19 in New Zealand and no evidence of community transfer. Although one-third of the participants had a COVID-19 test (only polymerase chain reaction tests were available), only one participant had been diagnosed with COVID-19. In 2022 (at the time of survey 2), there were reduced COVID restrictions owing to high vaccination rates; with Omicron, the infection rates have increased to 35% or more of the New Zealand population. Since now most people have had a COVID diagnosis, having a positive test will not be particularly discriminative. Therefore, we have modified those two items to “Have you ever been suspected of having COVID-19?” and “If yes, have you had a COVID test?” to capture stress owing to being a suspect of getting COVID-19 and stress of having a COVID test. In survey 1, other items also showed low-item-total correlations. These items were retained in the follow-up survey pragmatically and our findings from survey 2 affirm that those items showed acceptable reliability scores when the prevalence of COVID-19 changed considerably. In short, our team considered both psychometric and pragmatic considerations in designing the items of CPIS to reflect the fluid situation on the ground. 

In recognition of the positive impacts of COVID-19 [22,23,24], we added four items assessing the sense of the meaning to life. On reflection, these items appear limited in their assessment of this area. We are considering removing these items in future iterations of the CPIS to balance the question types and to reduce the response burden. This area can be assessed by a standardised scale such as the Post-traumatic Growth Inventory [45] to examine whether a person has undergone a positive transformation after a traumatic event, such as COVID-19. In addition, there were twelve opposite items in three domains (relationships, changes to daily routines and behaviours, changes to faith-related activities) of CPIS assessing the same idea but one from a positive, and the other from a negative direction. These items were added to conceptualize any change in these domains as being a stressor, whether it is positive or negative. Either of these pairs of items could be considered ‘stressful’, however, it is unlikely that participants would endorse both items, rather they would be more likely to endorse one, but not the other. Although these items were not highly correlated warranting item-response bias [46], we are considering removing them to reduce the response burden in future iterations of CPIS and to capture negative aspects of functioning in these domains alone by assessing conceptually similar items to shed light on the same underlying construct. The CPIS included two optional subscales assessing the stress owing to changes to education-related and faith-related activities in response to COVID-19. This paper demonstrates how to use CPIS with and without these subscales. Future researchers can consider taking out optional subscales depending on their research interests. Lastly, there can be other pandemic-related life events that can be stressful (such as stress owing to vaccination mandates, travel bans, etc.) or other positive life changes due to the pandemic (such as adopting a healthier lifestyle or improving financial status) which could be added in the future iterations of CPIS, if applicable to the pandemic context.

This study has the following strengths. The CPIS was developed using the same “participant researcher” approach applied to understanding the psychosocial impacts of the Canterbury earthquakes [25,26,34,35]. This approach assesses exposure to the immediate impacts of the disaster and the adverse personal, social, and economic consequences that follow. We believe that the use of pandemic-specific impact scales is required to identify specific impacts of COVID-related stressors, and this cannot be achieved by established measures alone. The CPIS offers some unique advantages over other available pandemic-related scales, including an assessment of both negative and positive impacts. The CPIS can also be used in the general population and targeted groups, such as healthcare workers, essential workers, and other at-risk groups. We have also demonstrated its use in different pandemic-related contexts.

This study has the following limitations. The main limitations include data collection from a non-representative New Zealand sample representing literate or high-income individuals. Since the psychological effects of COVID-19 were widely anticipated, it would be ideal to have a representative sample. The survey was delivered online, and evidence suggests that people living with disabilities (including mental illness) are less likely to participate in online surveys [47]. It would be interesting to explore the association between socio-demographic and study variables using regression analysis to pool predictor variables. This was not done to keep the emphasis on reporting the scale’s psychometric characteristics. Future analyses of this or an expanded data set using a regression analysis would be useful. 

Lastly, the COVID-19 mortality rate—even at its peak in New Zealand—was considerably lower than in many other countries. For instance, as of 3 March 2023, the USA’s total COVID-19 attributable death rate per 100,000 was estimated at 335.09, some 6.4 times higher than New Zealand’s rate of 52.71 [48]. This may affect the external validity and/or psychometric properties—especially in countries with considerably higher morbitiy and mortality rates. Although the CPIS was developed to be cross-culturally applicable, it has not yet been trialed in different cultures/countries—but consultation and co-design were used to fit it for such use. Further studies are underway in which a brief 32-item CPIS will be used in combination with other standardised measures of wellbeing (WHO-5), distress (K10, PTSD Checklist for DSM-5 [49]), and post-traumatic growth (PTGI) in several countries [50]. To achieve this, the CPIS and other measures were translated using parallel back-to-back translations by a team of proficient bilingual academics and researchers who were familiar with the content and context [51]. This follow-up study (Clinical Trials Registry (NCT05052333)) has the potential to contribute to an increased understanding of the psychosocial impacts of the COVID-19 pandemic and, importantly, to the development of psychosocial measures that allow cross-cultural comparisons. 

The COVID-19 pandemic has had a profound impact on the world, causing widespread illness, death, and disruption to daily life. The fear and uncertainty surrounding the virus, coupled with social isolation and financial insecurity, have contributed to increased rates of depression, anxiety, and other mental health issues. With vaccines now widely available, and case numbers decreasing in many parts of the world, it may seem that the worst of the pandemic is over. However, measures taken to examine the psychosocial impacts of the pandemic should not be disregarded as new mutations of the virus could potentially lead to another global crisis. It is important to continue exploring and addressing the psychosocial impacts of the current pandemic as it evolves by adapting pandemic-related scales if necessary in future waves, as well as making efforts to improve preparedness for potential future pandemics.

## 5. Conclusions

This paper reports on the development and validation of CPIS, a self-report measure that comprehensively examines the psychosocial impacts of the COVID-19 pandemic. Importantly, this psychometric study was carried out at two distinct time points using an online survey to capture different exposures to the pandemic in the New Zealand population. The newly developed scale was used in combination with established measures of psychological distress and wellbeing. Findings suggest a unidimensional structure within CPIS subscales and inter-relatedness among CPIS stress-related subscales. The CPIS effectively examines the psychosocial impacts, both positive and negative; while it correlates with psychological distress and a general wellbeing, it is not only a proxy for these constructs. The data were obtained from a non-representative New Zealand sample using an online survey. This is the main limitation of this study. Future researchers should explore the association between socio-demographic and study variables along with reporting the psychometric properties of CPIS. The CPIS was developed to be cross-culturally applicable, further work will examine its cross-cultural use in countries with much higher morbidity and mortality rates. It is imperative to sustain the efforts taken to investigate and address the psychosocial impacts of the COVID-19 pandemic while continuing to improve our readiness for future pandemics.

## Figures and Tables

**Table 1:** Showing Items, Key Domains, and Subscales of the COVID Psychosocial Impacts Scale (CPIS).

Key Domains		Subscale
Personal Impacts		Personal stress
	1. Do you have an underlying health condition that could make you vulnerable to COVID-19 (e.g., heart disease, difficulty breathing, weakened immunity or cancer)?	
	2. Have you had a COVID-19 test? (Have you ever been suspected of having COVID-19?) *	
	3. If Yes, was the result positive? (If yes, have you had a COVID-19 test?) *	
	4. Have you felt at risk of being exposed to someone with COVID-19?	
Family Impacts		Social stress
	5. Does any family member have an underlying health condition that could make them vulnerable to COVID-19 (e.g., heart disease, difficulty breathing, weakened immunity, or cancer?)	
	6. Do you feel any family member is at risk of being exposed to someone with COVID-19 through their work?	
	7. Has any family member had a COVID-19 test? (Has any family member been suspected of having COVID-19?) *	
	8. If yes, was the result positive?	
	9. Do you have family overseas who you are worried are at risk of getting COVID-19?	
	10. Do you have family overseas who you are not able to visit or who cannot visit you?	
Living Arrangement		Social stress
	11. How much stress is this living arrangement causing you?	
Work and COVID-19		Financial stress
	12. Are you considered an essential worker (e.g., in healthcare, law enforcement, emergency services, or provider of essential goods/services)?	
	13. Do you have a close friend or family member who is an essential worker?	
Education		Education-related stress
	14. Had to change the mode of study (e.g., online).	
	15. Had access to a quiet space to study.	
	16. Had access to adequate computing facilities (e.g., personal laptop or computer, internet, etc.).	
	17. Are you concerned about its impact on your grades?	
	18. Are you concerned about its impact on your future employment?	
Employment		Financial stress
	19. Had a major change in working hours?	
	20. Had to change the place of work to home?	
	21. Had to change to a different type of work?	
	22. Have you or anyone in your household lost their job?	
	23. Is a business you or someone in your household owns or works in under threat of survival?	
Income		Financial stress
	24. Has your household had a major deterioration in financial circumstances?	
	25. Has your household had a major deterioration in your ability to pay your rent or mortgage?	
	26. Has your household had a major deterioration in your ability to pay your bills or for food?	
	27. Has your household needed to seek help from a food bank or other charitable organisation?	
Relationships		Social stress
	28. Has it increased difficulty and tension in the family?	
	29. Has it brought the family closer together?	
	30. Has this decreased your contact with friends (physical or online)?	
	31. Has this increased your contact with friends (physical or online)?	
Changes to daily routines and behaviours		Personal stress
	32. Has the COVID-19 pandemic led to changes in your daily routine (for example, sleep, exercise, free time, pleasurable activities, or hobbies)?	
	33. Has this led to you being less involved in family life?	
	34. Has this led to you being more involved in family life?	
	35. Has this caused you to delay/avoid seeking health care when you or a family member needed it?	
	36. Has this increased your contact with healthcare for you or a family member?	
	37. Have there been any other major changes in routine?	
Changes to faith-related activities		Faith-related stress
	38. Has this decreased your involvement with religious activities (in person or online)?	
	39. Has this increased your involvement with religious activities (in person or online)?	
	40. Has this decreased your connection with religious social gatherings (in person or online)?	
	41. Has this increased your connection with religious social gatherings (in person or online)?	
Exposure to information about COVID-19		Personal stress
	42. How much stress is the news about COVID-19 causing you?	
Comparing now with before the COVID-19 pandemic		Perception of overall life stress pre/post COVID-19
	43. How would you rate your personal level of stress?	
	44. How would you rate the overall stress in your household?	
	45. How would you rate your personal psychological wellbeing?	
	46. How would you rate the overall psychological wellbeing of your household members?	
Silver Lining		Positive Impacts
	47. Made me value family time more.	
	48. Made me realise what was important in life.	
	49. Led to an increased connection to my faith.	
	50. Gave me a greater sense of purpose.	

* Note: Three items (items 2, 3, and 7) were modified in survey 2 owing to psychometric and pragmatic considerations. The items in survey 2 are shown in brackets.

**Table 2:** Showing Exploratory Factor Analysis ^1^ on the Core Subscales of COVID Psychosocial Impacts Scale (CPIS).

Subscale	Survey 1 (N = 663)			Survey 2 (N = 687)		
	Mean (SD)	Factor 1	Factor 2	Mean (SD)	Factor 1	Factor 2
Personal stress	10.4 (6.9)	**0.87**	−0.08	13.9 (7.6)	**0.89**	−0.07
Social stress	12.8 (6.9)	**0.84**	−0.06	15 (8.5)	**0.86**	−0.10
Financial stress	5.7 (6.6)	**0.67**	−0.02	4 (4.6)	**0.68**	−0.07
Perception of overall life stress pre/post COVID-19	13.0 (2.6)	**0.71**	0.18	20 (12.7)	**0.68**	**0.34**
Positive impacts	10.6 (4.4)	0.01	**0.99**	15 (10.1)	0.01	**0.95**

^1^ The extraction method is principal component analysis. Components in bold have an *r* value above 0.30.

**Table 3:** Showing Descriptive Statistics on COVID Psychosocial Impacts Scale (CPIS), Kessler 10 (K10), and World Health Organization Well-Being Index 5 (WHO-5).

	Survey 1		Survey 2	
	Score Range	Mean (SD)	Score Range	Mean (SD)
CPIS composite score	15–147	51.8 (18.2)	11–152	56.3 (20.4)
K10	10–50	18.5 (7.3)	10–43	16.5 (6.6)
WHO-5	0–100	52.3 (21.3)	0–100	57.1 (23.6)
CPIS total score including Education-related stress subscale	21–118	62.8 (21.7)	30–169	69.4 (24.4)
K10	10–49	19.9 (7.2)	10–41	19.9 (7.6)
WHO-5	4–88	49.6 (20.5)	0–100	52.7 (22)
CPIS total score including Faith-related stress subscale	27–148	63.1 (22.5)	19–172	61.8 (22.7)
K10	10–45	19.1 (7)	10–39	16.7 (6.5)
WHO-5	4–92	49.8 (21.3)	8–100	55.4 (22.4)

**Table 4:** Showing Construct Validity of COVID Psychosocial Impacts Scale (CPIS) and CPIS Subscales with Kessler 10 (K10) and World Health Organization Well-Being Index 5 (WHO-5) ^1^.

	Personal Stress	Social Stress	Financial Stress	Perception of Overall Life Stress Pre/Post COVID-19	Positive Impacts	CPIS Composite Score	K10	WHO-5
Personal stress	1	0.75 **	0.47 **	0.46 **	−0.13 **	0.87 **	0.55 **	−0.47 **
Social stress	0.67 **	1	0.40 **	0.43 **	−0.14 **	0.86 **	0.51 **	−0.42 **
Financial stress	0.50 **	0.38 **	1	0.33 **	−0.04	0.69 **	0.29 **	−0.27 **
Perception of overall life stress pre/post COVID-19	0.46 **	0.49 **	0.28 **	1	0.07	0.61 **	0.50 **	−0.51 **
Positive impacts	−0.03	−0.03	0.01	0.08	1	0.08 *	−0.11 **	0.02
CPIS composite score	0.84 **	0.82 **	0.72 **	0.61 **	0.22 **	1	0.56 **	−0.50 **
K10	0.54 **	0.50 **	0.30 **	0.54 **	0.03	0.57 **	1	−0.75 **
WHO-5	−0.42 **	−0.38 **	−0.23 **	−0.52 **	−0.14 **	−0.48 **	−0.71 **	1

^1^ Data from survey 1 (N = 663) is shown in blue and data from survey 2 (N = 687) is in grey. ** Correlation is significant at the 0.01 level (2-tailed). * Correlation is significant at the 0.05 level (2-tailed).

**Table 5:** Showing Descriptive Statistics on COVID Psychosocial Impacts Scale (CPIS), CPIS Core Subscales, Kessler 10 (K10), and World Health Organization Well-Being Index 5 (WHO-5) on Subsample (n = 271).

	Survey 1 Mean (SD)	Survey 2 Mean (SD)	Mean Difference [95% CI]
Personal stress	10.1 (5.9)	15.2 (7.4)	5.2 [4.4, 5.9]
Social stress	10.7 (6)	16.3 (8.6)	5.6 [4.7, 6.5]
Financial stress	4.9 (5.9)	4 (5.4)	−0.9 [−1.7, −0.1]
Perception of overall life stress pre/post COVID-19	12.9 (2.5)	12.9 (2.9)	0 [−0.4, 0.4]
Positive impacts	11 (4.1)	9.9 (3.6)	−1.1 [−1.6, −0.5]
CPIS composite score	49.6 (15.9)	58.3 (19.5)	8.8 [6.7, 10.8]
K10	16.1 (26.3)	17.8 (7.1)	1.7 [−1.4, 4.8]
WHO-5	53 (22.1)	54 (23)	1 [−1.3, 3.3]

**Figure A1:**
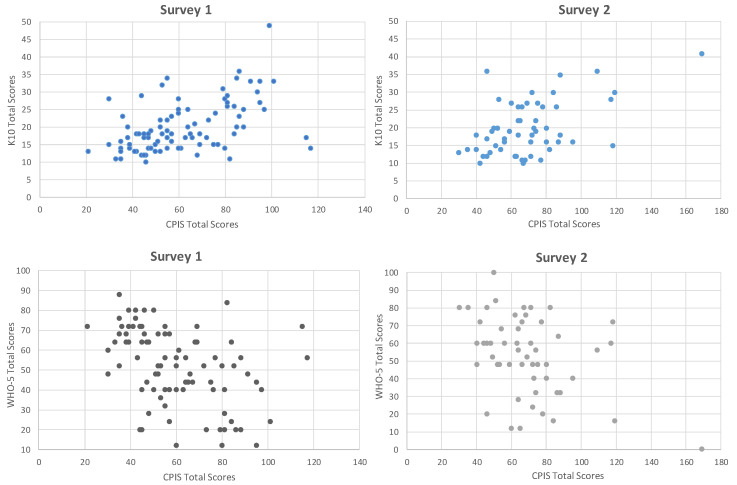
Showing Scatter Plot Showing a Positive Linear Correlation between COVID Psychosocial Impacts Scale (CPIS) and Kessler 10 (K10); and a Negative Linear Correlation between CPIS and World Health Organization Well-Being Index 5 (WHO-5) for Participants Who Responded to the Education-related Stress Subscale.

**Figure A2:**
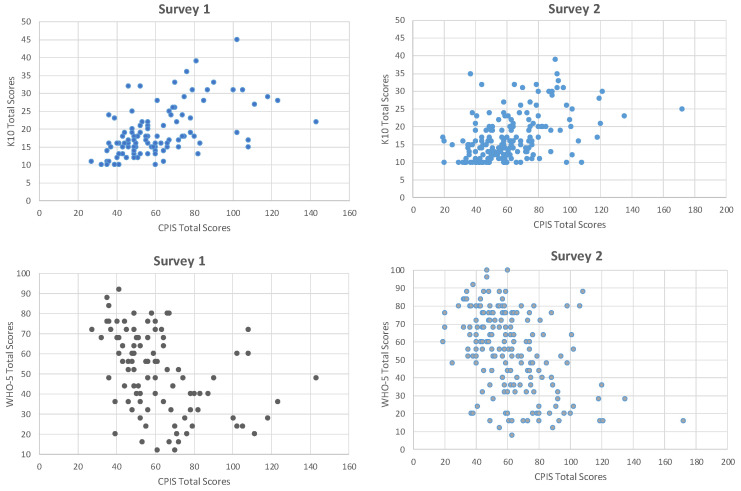
Showing Scatter Plot Showing a Positive Linear Correlation between COVID Psychosocial Impacts Scale (CPIS) and Kessler 10 (K10); and a Negative Linear Correlation between CPIS and World Health Organization Well-Being Index 5 (WHO-5) for Participants Who Responded to Faith-related Stress Subscale.

## Data Availability

Data are available on request.

## Appendix A

**Table A1:** Showing Demographic Characteristics of Participants.

Demographics	Survey 1 (N = 663)		Survey 2 (N = 687)	
	n	%	n	%
Gender				
Male	155	23	314	46
Female	497	75	364	53
Other	11	2	9	1
Age range (in years)				
18–19	8	1	0	0
20–29	87	13	32	5
30–39	140	21	71	10
40–49	221	33	139	20
50–59	107	16	167	24
60–69	71	11	156	23
70 and above	29	4	122	18
Marital status				
Single	119	18	95	14
In relationship	153	23	123	18
Married	341	51	398	58
Separated	12	2	23	3
Divorced	28	4	27	4
Widowed	10	2	21	3
Ethnicity				
NZ European	583	88	613	89
Māori	44	7	38	6
Pacific peoples	6	1	6	1
Asian	10	2	22	3
Middle Eastern/Latin American/African	4	1	4	1
Other	69	10	52	8
Highest education level				
No formal education	5	1	10	1
Secondary school	50	8	53	8
Tertiary qualification	109	16	141	21
Bachelor degree	216	33	216	31
Postgraduate degree	283	43	267	39
Current work or labour force status				
Employed full-time	361	54	356	52
Employed part-time	171	26	139	20
Unemployed	43	7	28	4
Not in the labour force	90	14	164	24
Occupation				
Manager	80	12	96	14
Professional	331	50	326	47
Technicians and trade workers	30	5	23	3
Community and personal service workers	21	3	18	3
Clerical and administration workers	49	7	33	5
Sales workers	11	2	7	1
Machinery operators and drivers	2	0	4	1
Labourers	2	0	4	1
Residential categories/Others	137	21	176	26
Annual family income				
$14,000 or less	84	13	58	8
$14,001–$48,000	156	24	173	25
$48,001–$70,000	126	19	113	16
>$70,000	295	45	343	50
Trauma exposure before COVID-19				
Natural disaster	225	34	249	36
Living in a war zone or exposed to military conflict	17	3	31	5
Childhood adversity before the age of 16	164	25	153	22
Physical or sexual assault after the age of 16	84	13	68	10
Serious physical accident	58	9	73	11
Other	196	30	64	9
COVID-19 Specific Demographics				
Current living arrangement				
Living alone	77	12	106	15
Partner/Spouse	203	31	302	44
Family	310	47	250	36
Flatmates/Friends	66	10	28	4
Hall of Residence/Hostel	6	1	1	0
Change in living arrangement since COVID-19				
No change	583	88	580	84
Increased number of people	37	6	51	7
Decreased number of people	43	7	56	8
Sources of information regarding COVID-19				
News	603	91	571	83
Facebook/Twitter	268	40	141	21
Health websites	411	62	457	67
Word of mouth	154	23	174	25
Others	94	14	158	23
Number of hours seeking information about COVID-19				
Less than one hour per day	505	76	602	88
1–2 h per day	135	20	70	10
2–4 h per day	17	3	11	2
4–8 h per day	5	1	3	0
More than 8 h per day	0	0	1	0
The participant is an essential worker	154	23	170	25
Close Family and friends are an essential worker(s)	221	33	375	55

## Appendix B

**Table A2:** Showing Exploratory Factor Analysis ^1^ of COVID Psychosocial Impacts Scale Subscales for Participants Who Responded on Education-related Stress Subscale.

Subscales	Survey 1 (n = 91)			Survey 2 (n = 55)		
	Mean (SD)	Factor 1 ^a^	Factor 2	Mean (SD)	Factor 1 ^a^	Factor 2
Personal stress	10.7 (5.7)	**0.81**	−0.17	16.6 (8)	**0.87**	−0.07
Family stress	12.7 (6.1)	**0.83**	0.14	18 (9.8)	**0.82**	−0.24
Financial stress	6.7 (7.1)	**0.61**	−0.59	7.3 (7.5)	**0.58**	**0.43**
Perception of overall life stress pre/post COVID-19	13.5 (2.5)	**0.76**	0.12	13.7 (2.6)	**0.61**	**0.49**
Education-related stress	6.9 (5.3)	**0.71**	0.19	4.5 (5)	**0.66**	−0.28
Positive impacts	11 (4.4)	0.19	**0.85**	9.3 (3.2)	−0.14	**0.74**

^1^ The extraction method is principal component analysis. Components in bold have an *r* value above 0.3. ^a^ Factor having an α-value above 0.70.

**Table A3:** Showing Exploratory Factor Analysis ^1^ of COVID Psychosocial Impacts Scale Subscales for Participants Who Responded on Faith-related Stress Subscale.

Subscales	Survey 1 (n = 103)			Survey 2 (n = 181)		
	Mean (SD)	Factor 1 ^a^	Factor 2	Mean (SD)	Factor 1 ^a^	Factor 2
Personal stress	11.7 (7.3)	**0.90**	−0.05	14.2 (7.6)	**0.88**	−0.16
Family stress	12.6 (7.1)	**0.88**	−0.09	15 (8.7)	**0.84**	−0.10
Financial stress	7.4 (8)	**0.72**	0.06	5.2 (6.4)	**0.64**	−0.08
Perception of overall life stress pre/post COVID-19	13.1 (2.7)	**0.65**	0.33	12.8 (3)	**0.66**	0.02
Faith-related stress	4.3 (3.1)	**0.45**	−0.12	4.3 (3.9)	**0.50**	**0.42**
Positive impacts	12.6 (3.7)	−0.09	**0.95**	10 (3.8)	0.06	**0.93**

^1^ The extraction method is principal component analysis. Components in bold have an *r* value above 0.3. ^a^ Factor having an α-value above 0.70.

## Appendix C

**Table A4:** Showing the Descriptive Statistics of the COVID Psychosocial Impacts Scale (CPIS), CPIS Subscales, Kessler 10 (K10), and World Health Organization Well-Being Index 5 (WHO-5) for Participants who responded to Both of the Optional Subscales of CPIS.

	Survey 1 (n = 13)		Survey 2 (n = 13)	
	Score Range	Mean (SD)	Score Range	Mean (SD)
Personal stress	4–25	12.1 (6.4)	7–35	15.9 (6.6)
Social stress	5–25	14.1 (5.8)	6–45	18.6 (10.8)
Financial stress	0–35	6 (9.7)	0–22	8.7 (6.5)
Perception of overall life stress pre/post COVID-19	12–17	13.8 (1.6)	10–19	13.6 (2.4)
Education-related stress	1–16	8.3 (5)	0–13	4.5 (4.7)
Faith-related stress	2–13	4.5 (3.2)	0–8	3.3 (2.3)
Positive impacts	4–20	12.7 (4.6)	5–15	10.4 (2.9)
CPIS total score	46–121	71.5 (22.8)	43–121	75.1 (19)
K10	12–36	21.2 (8)	10–36	16.4 (6.6)
WHO-5	20–80	48.9 (22.1)	20–100	65.9 (20.2)

## Appendix D

**Table A5:** Showing Construct Validity of COVID Psychosocial Impacts Scale (CPIS) and CPIS Subscales with Kessler 10 (K10) and World Health Organization Well-Being Index 5 (WHO-5) for Participants Who Responded on Education-related Stress Subscale ^1^.

	Personal Stress	Social Stress	Financial Stress	Perception of Overall Life Stress Pre/Post COVID-19	Education-Related Stress	Positive Impacts	CPIS Total Score	K10	WHO-5
Personal stress	1	0.74 **	0.39 **	0.41 **	0.46 **	−0.09	0.87 **	0.51 **	−0.45 **
Social stress	0.57 **	1	0.22	0.33 *	0.48 **	−0.13	0.83 **	0.47 **	−0.41 **
Financial stress	0.55 **	0.38 **	1	0.42 **	0.25	−0.01	0.69 **	0.17	−0.10
Perception of overall life stress pre/post COVID-19	0.46 **	0.53 **	0.31 **	1	0.18	0.05	0.55 **	0.32 *	−0.49 **
Education-related stress	0.42 **	0.53 **	0.21 *	0.50 **	1	−0.11	0.63 **	0.39 **	−0.16
Positive impacts	0.10	0.22 *	−0.16	0.14	0.11	1	0.03	−0.12	0.06
CPIS total score	0.81 **	0.82 **	0.66 **	0.66 **	0.68 **	0.29 **	1	0.51 **	−0.42 **
K10	0.35 **	0.40 **	0.21 *	0.35 **	0.55 **	0.16	0.50 **	1	−0.73 **
WHO-5	−0.39 **	−0.36 **	−0.25 *	−0.45 **	−0.38 **	−0.20	−0.48 **	−0.76 **	1

^1^ Data from survey 1 (n = 91) is shown in blue and data from survey 2 (n = 55) is in grey. ** Correlation is significant at the 0.01 level (2-tailed). * Correlation is significant at the 0.05 level (2-tailed).

## Appendix E

**Table A6:** Showing Construct Validity of COVID Psychosocial Impacts Scale (CPIS) and CPIS Subscales with Kessler 10 (K10) and World Health Organization Well-Being Index 5 (WHO-5) for Participants Who Responded on Faith-related Stress Subscale ^1^.

	Personal Stress	Social Stress	Financial Stress	Perception of Overall Life Stress Pre/Post COVID-19	Faith-Related Stress	Positive Impacts	CPIS Total Score	K10	WHO-5
Personal stress	1	0.75 **	0.44 **	0.47 **	0.34 **	−0.06	0.86 **	0.43 **	−0.42 **
Social stress	0.79 **	1	0.35 **	0.48 **	0.26 **	0.02	0.85 **	0.40 **	−0.39 **
Financial stress	0.604 **	0.47 **	1	0.28 **	0.30 **	−0.03	0.65 **	0.26 **	−0.26 **
Perception of overall life stress pre/post COVID-19	0.45 **	0.46 **	0.37 **	1	0.09	0.12	0.59 **	0.49 **	−0.47 **
Faith-related stress	0.32 **	0.38 **	0.12	0.11	1	0.15 *	0.50 **	0.08	−0.06
Positive impacts	−0.08	−0.11	−0.04	0.07	0.00	1	0.19 *	−0.05	0.02
CPIS total score	0.89 **	0.85 **	0.77 **	0.58 **	0.43 **	0.10	1	0.45 **	−0.43 **
K10	0.55 **	0.48 **	0.29 **	0.55 **	0.23 *	−0.05	0.53 **	1	−0.74 **
WHO-5	−0.38 **	−0.34 **	−0.25 **	−0.48 **	−0.18	−0.18	−0.44 **	−0.70 **	1

^1^ Data from survey 1 (n = 103) is shown in blue and data from survey 2 (n = 181) is in grey. ** Correlation is significant at the 0.01 level (2-tailed).* Correlation is significant at the 0.05 level (2-tailed).
